# Editorial to Special Issue—“Structure-Activity Relationships (SAR) of Natural Products”

**DOI:** 10.3390/molecules26020250

**Published:** 2021-01-06

**Authors:** Wolfgang Sippl, Fidele Ntie-Kang

**Affiliations:** 1 Institute of Pharmacy, Martin-Luther University of Halle-Wittenberg, Kurt-Mothes-Str. 3, 06122 Halle, Germany; wolfgang.sippl@pharmazie.uni-halle.de; 2 Department of Chemistry, Faculty of Science, University of Buea, P.O. Box 63, Buea CM-00237, Cameroon; 3 Institute of Botany, Technical University of Dresden, Zellescher Weg 20b, 01062 Dresden, Germany

The topic of structure-activity-relationships (SAR) has recently drawn a lot of attention, and there is increasing interest in natural products (NPs) as a “source of inspiration” for the discovery of new lead compounds [1,2]. A quick search of the terms “natural product” and “structure-activity-relationships” in the PubMed database [3], for example, yielded ~675,000 and 210,000 hits, respectively. A search of the term “secondary metabolite”, the less frequently employed term for NPs, in the same database yielded ~28,000 hits. First of all, compounds from natural sources have been fine-tuned over the ages to bind to specific classes of drug targets [4,5]. Thus, NPs are gaining attention as sources of lead compounds with novel scaffolds. Secondly, the relationship between chemical structure and the biological activities of compounds have often been investigated, with the view of finding out what chemical modification on the structure would improve the activity and/or reduce the toxic effects of compounds [4,5].

In this Special Issue (SI), we focus on NPs and NP mimics and their derivatives, which play a role in several biochemical processes, including those showing potential for development into drugs [6]. The goal was to investigate compounds from biological sources (including fungal, microbial, marine, and plant sources), which host biosynthesis processes for making secondary metabolites. Although synthetically modified compounds (semi-synthetic NPs or pseudo-NPs) were also welcome in this SI, emphasis was on those derived from QSAR or SAR studies through computer modeling, in vitro and in vivo testing. The current SI is the result of a call that was sent out during mid-2019 and which resulted in several submissions, out of which nine papers (6 original research articles and 3 reviews) appeared in the final published version.

This SI is a follow up of a previously dedicated SI to this subject in the same journal [7]. The topic SAR of NPs is quite broad in scope and could include areas like; bioactive NPs from diverse sources (including those of marine and terrestrial origins) with biological activities exploitable for the treatment of diverse diseases (including infectious diseases and cancer), e.g., mezzettiaside NPs and their disaccharide analogues are known to exhibit both anticancer (H460) and antibacterial (*Bacillus subtilis*) activities, and their SAR have been previously investigated [8]. Topics that provide insight from binding free energy calculations, docking, pharmacophore modeling, and molecular dynamics involving small molecule NPs and NP mimics are also included [9], along with quantitative structure-activity-relationships (QSAR) applications [10], and may also extend to natural remedies and the pharmacogenomics of age-related and neurodegenerative disorders [11], as well as their the implications in disease-associated epigenetic mechanisms and the chemoinformatic exploration of the chemical space and target space of natural products with epigenetic functions [12]. In terms of applications beyond drug discovery, the role of NPs in environmental remediation could as well be considered [13], as well as the epigenetic mechanisms that lead to the ecological balance between plants, fungi, and microbes, resulting in the biosynthesis of specific kinds of secondary metabolites [14,15]. More advanced topics could include polypharmacology and its association with treatment using natural remedies [16], as well as protein glycosylation and implications for genes, environment, and plant and human diseases [17,18].

Case studies in the literature include SAR studies on NPs which have led to the synthesis of potent analogues, e.g., a recent review has presented diverse SAR studies which have led to the discovery of biologically useful analogues of macrocyclic NPs, illustrating the importance of designing building blocks accompanied by synthetic strategies [19]. To illustrate this concept, Xiong et al. have investigated the signaling of synthetic analogues of the cyclic depsipeptide YM-254890, and the structurally similar FR900359, by investigating the SAR of the compounds and their synthetic analogues in G protein-coupled receptor (GPCR) signaling pathways [20]. Other examples include SAR investigations of synthetic coumarin derivatives showing anti-tubercular activities [21] and the use of SAR for the investigation of the anticancer activities of marine-derived bioactive compounds [22].

The topics published in the current SI were quite diverse, touching the different areas highlighted in the previous paragraphs, e.g., molecular docking combined with pharmacophore-based virtual screening have led to the identification of the hydrolysable tannin NP, chebulagic acid, as a moderately active inhibitor of the influenza A virus ion channel [23]. This study by Duncan et al. could further explain why the NP only showed experimental activity against the mutant, and not the wild type influenza virus. While Jöhrer et al. were able to show that the position C-25 of a series of terpenooids from Black Cohosh (*Actaearacemosa*) could increase their cytotoxic effects, the arabinose moiety at position C-3 having an impact on this activity, Babiaka et al. used molecular docking of mono- and bis-indole alkaloids towards a homology model of *Onchocerca ochengi* thioredoxin reductase to attempt an explanation of the anti-filarial activities of these compounds [24]. Kumar et al. combined several in vitro, in silico, and in vivo experiments to investigate the anti-anaphylactoid activity of Genistein in order to understand the possible molecular mechanisms of its action, which is mediated by a family of GPCRs [25]. Meanwhile, Wurzlbauer et al. were able to show, through an investigation of over 60 analogues of the β-carboline alkaloid harmine, that “beneficial” residues at the C-1 position (occupied by methyl or chlorine) could make the difference between the desired kinase (DYRK1A) inhibition and the non-desired monoamine oxidase A inhibition by extensive docking of the two protein targets [26]. Another study involved an attempt to reduce the cytotoxic activity of waste derived from *Chenopodium quinoa* husks by microwave-assisted hydrolysis reaction to produce monodesmosidic saponins from the husk extract in an optimal manner using mass spectrometry. Colson et al. were able to use the IMPALA in silico procedure to verify that the monodesmosidic saponins interacted preferably with a model phospholipid bilayer. This could provide an explanation why the measured hemolytic activity increased experimentally during the microwave hydrolytic process [27].

Review articles in this SI discuss recent studies that attempt to investigate the SAR of NPs that have shown anti-HIV activities [28], modulatory activities in zinc-independent histone deacetylases, sirtuins often implicated in human disease [29] and SAR in plant-derived sweeteners [30]. The readers are invited to enjoy the reading of the articles of this SI.

## Data Availability

No new data were created or analyzed in this study. Data sharing is not applicable to this article.
